# Curvature-dependent constraints drive remodeling of epithelia

**DOI:** 10.1242/jcs.222372

**Published:** 2019-01-24

**Authors:** Florian A. Maechler, Cédric Allier, Aurélien Roux, Caterina Tomba

**Affiliations:** 1 Department of Biochemistry, University of Geneva, CH-1211 Geneva, Switzerland; 2 CEA, LETI, DTBS, LISA, Université Grenoble Alpes, F-38000 Grenoble, France; 3 NCCR Chemical Biology, University of Geneva, CH-1211 Geneva, Switzerland

**Keywords:** 3D cell culture, Bioengineering, Cylindrical confinement, Epithelial morphogenesis

## Abstract

Epithelial tissues function as barriers that separate the organism from the environment. They usually have highly curved shapes, such as tubules or cysts. However, the processes by which the geometry of the environment and the cell's mechanical properties set the epithelium shape are not yet known. In this study, we encapsulated two epithelial cell lines, MDCK and J3B1A, into hollow alginate tubes and grew them under cylindrical confinement forming a complete monolayer. MDCK monolayers detached from the alginate shell at a constant rate, whereas J3B1A monolayers detached at a low rate unless the tube radius was reduced. We showed that this detachment is driven by contractile stresses in the epithelium and can be enhanced by local curvature. This allows us to conclude that J3B1A cells exhibit smaller contractility than MDCK cells. Monolayers inside curved tubes detach at a higher rate on the outside of a curve, confirming that detachment is driven by contraction.

## Abstract (precis)

**Summary:** Epithelia growing under tubular confinement are shown to detach and constrict to form a narrow tube in a process that depends on curvature of the substrate, cell shape and contractility.

## INTRODUCTION

In metazoan animals, epithelial tubules are structural features of organs that serve essential functions such as the transport of gases, liquids, metabolites or cells. Also, during embryogenesis, epithelial tubules are often formed as intermediate templates for organs. For example, gastrulation or neurulation are processes in which epithelia deform into tubules to create the gut and the central nervous system, respectively. The mechanisms underlying the formation of organs and epithelial tubes during embryogenesis are difficult to unravel because of the complexity of entirely controlling (genetically, biochemically and mechanically) the microenvironment of these structures. This morphogenesis relies on complex spatial rearrangement leading to complex structures (Keller, 2002; Salazar-Ciudad et al., 2003). The use of tissue engineering methods could considerably simplify the task by controlling at least the mechanical and the biochemical microenvironment of these tubes. This would help in deciphering which cellular properties dictate the final shape of the tissue grown in a chemically and mechanically controlled microenvironment.

Cells interact with their specific microenvironment, which, *in vivo*, has a complex three-dimensional architecture (Alford and Taylor-Papadimitriou, 1996; Broders-Bondon et al., 2018). Previously, *in vitro* studies of cell monolayer cultures were performed on flat (2D) substrates, neglecting the possible effect of the three-dimensional (3D) architecture of living tissues. A 2D culture can consequently neither support the tissue-specific functions of most cell types nor properly predict *in vivo* tissue functions that may rely on geometry (Greek and Menache, 2013).

To recapitulate a functional 3D organization, a simple method has been to culture specific cell types in hydrogels made from components of the extracellular matrix (ECM) (Caliari et al., 2016). The interactions between cells and the ECM hydrogel create a complex network of mechanical and biochemical signals that are critical for normal cell physiology (Abbott, 2003; Griffith and Swartz, 2006; Pampaloni et al., 2007). However, the mechanical properties of such gels, as well as their precise chemical composition, are difficult to control or/and change (Beduer et al., 2015; Benenson and Lutolf, 2017). This has prompted the use of artificial hydrogels in which composition and stiffness can be controlled accurately (Gjorevski et al., 2016). However, this method usually fails to apply geometrical or shape constraints on the growing tissue, as is the case *in vivo*, where tissues grow under the pressure of surrounding organs. A growing effort is being made to design assays in which tissues are grown in microenvironments of controlled shape (Burute et al., 2017; Laurent et al., 2017). In accordance with this, the use of microfluidics in 3D culture assays has become a standard technique in obtaining a controlled microenvironment which incorporates different cell types, respects the cell shape and mimics the mechanical and chemical diversity of living tissues with greater accuracy (van Duinen et al., 2015).

Here, we present a cylindrical cell container made of alginate, in which the cells were grown in a tube of similar dimensions to that of many *in vivo* tubular structures. The encapsulation technique used to produce these tubes has already proved itself useful by producing hollow spheres to study the mechanics of tumor growth (Alessandri et al., 2013). In these hollow spheres, coated with Matrigel (a commercial ECM extract), neuronal stem cells can be differentiated into neurospheres, which are protected by the alginate shell, allowing for their manipulation (Alessandri et al., 2016). This technique controls many constraints that could impact epithelial morphogenesis and helps decipher the specific impact of the microenvironment on cell growth, as well as tissue response to physical constraints (Roskelley et al., 1995).

With this cell container, we aim to understand how the cylindrical shape constraining growth could affect the global organization and final shape of two kinds of epithelial cell monolayers. We have selected two cell lines for their ability to form well-organized epithelial layers, but with different cell size and appearance: Madin–Darby canine kidney cells (MDCK) and EpH4-J3B1A mammary gland epithelial cells (J3B1A). Both are among the few cell lines that generate tubular structures in 3D cell cultures (Soulié et al., 2014). MDCK cells are a model cell type in tissue mechanics and collective migration that form monolayers with a relatively homogeneous cell aspect ratio. MDCK cells are able to form cysts, i.e. spherical and polarized monolayers with an inner lumen, from which tubulogenesis is induced when exposed, for example, to hepatocyte growth factor (O'Brien et al., 2002). J3B1A cells show slightly larger dimensions and have a more squamous cell aspect (Soulié et al., 2014). They usually form spheroidal cysts as well, but exhibit branching tubules in the presence of low concentrations of transforming growth factor beta (Montesano et al., 2007).

## RESULTS

### MDCK and J3B1A cells adapt their initial growth under tubular confinement

**Fig. 1.**
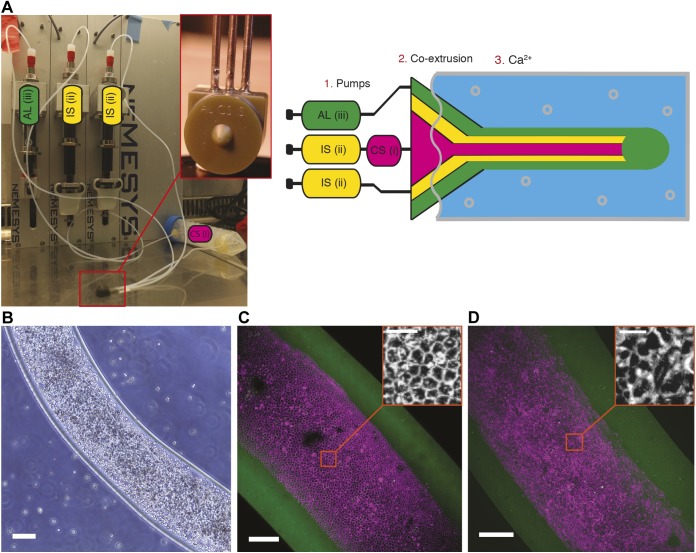
**Set-up for the production of alginate tubes coated with a Matrigel layer on the inner side of the shell, where epithelial cells form a monolayer.** (A) Photo (left) and diagram (right) of the set-up. 1. Pumps: set of three syringes controlling the flow of the cell solution [CS (i)], the sorbitol [IS (ii)] and the alginate [AL (iii)]. 2. Co-extrusion: 3D printed device (photograph in the inset). 3. Ca^2+^: calcium gelation bath. (B) Bright-field image of a tube of MDCK cells at day 0. Scale bar: 200 µm. (C,D) Maximum-intensity projections of 3D confocal images of a tube of MDCK cells (20% Matrigel, day 5) (C) and J3B1A cells (40% Matrigel, day 8) (D). Alginate tube, green; cell membrane, magenta (CellMask). Scale bars: 100 µm (20 µm, insets).

In this study, we confined and grew MDCK and J3B1A cell lines into a biocompatible and viscoelastic hollow tube made of alginate, a permeable (cut-off is 100 kDa) polymer with high potentials in biomaterials (Augst et al., 2006). Using 3D-printed microfluidic chips, a co-axial three-layered jet flow was injected into a calcium bath (Fig. 1A). The microfluidic chip is a 3D-printed device connecting three entry channels. These entries receive a flow from the connected syringe, respectively (i) a mix of cells, Matrigel and sorbitol (CS), (ii) sorbitol (IS) and (iii) alginate (AL). Using low-speed flow in the syringes allows the formation of droplets at the exit point of the microfluidic device; these then fall into the calcium bath at 37°C causing the alginate to polymerize into capsules (Alessandri et al., 2016). However, when the fluxes of the syringes were appropriately increased and the nozzle was immerged in the calcium bath, the resulting continuous jet at the exit point of the chip produced a cylindrically constrained environment for the cells. A single tube can be elongated until the alginate stock is fully used, which corresponds to several meters of tubes. In this study, the length of the tubes filled with cells was at least a few centimeters in order to ensure an aspect ratio (length:radius) of about 100, avoiding eventual effects due to the tube's ends. Two different microfluidic devices were designed in this study with exit canals of a diameter of 200 µm and 120 µm. For example, with J3B1A cells and a 30% concentration of Matrigel these devices produced tubes with inner diameters of 428±24 µm (defined as ‘regular tubes’) and 269±13 µm (‘small tubes’), respectively (mean±s.d., here and henceforth unless otherwise noted). These diameters are smaller than the size at which diffusion of gases and nutrients starts to be limiting (typically 500 µm) (Freyer and Sutherland, 1986).

Since epithelial cells require efficient adhesion to their substrate to properly develop and since alginate does not provide a correct cell-adhesive interface (Lee and Mooney, 2012), a thin layer of Matrigel coating the inner surface of the hollow alginate tubes is required. This layer was prepared by adding Matrigel to the cold cell solution (4°C) and co-polymerized with the alginate shell in the calcium bath (37°C) (Alessandri et al., 2016). This led to the confining of cells in a tubular enclosure, where cells initially flowed freely inside the lumen of the tube. Then, they adhered to the Matrigel coating on the inner surface of the alginate wall (Fig. 1B). Through proliferation, both cell lines, MDCK (Fig. 1C) and J3B1A (Fig. 1D) cells, formed clusters that merged into a monolayer, which closed upon reaching confluence. The tissue thus formed a cylindrical monolayer of cells with a lumen, with dimensions imposed by the alginate shell. Moreover, MDCK cells were more regularly organized when compared to J3B1A cells, which may lead to generation of higher forces in the tissue (Fig. 1C,D, insets). We deeply explored this observation by first characterizing the dynamics of proliferation of MDCK and J3B1A cells into these alginate tubes.

### Three main stages characterize epithelial growth in alginate tubes

**Fig. 2.**
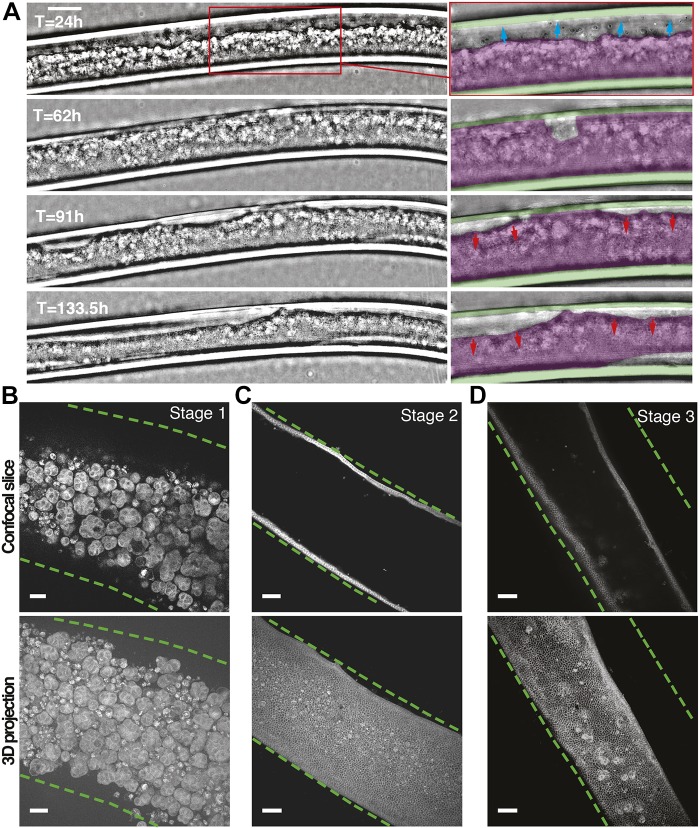
**MDCK cell monolayer growth in alginate tubes.** (A) Live imaging performed across a 5.5 day range with lens-free microscopy (phase images). Frames in the insets show monolayer progression (blue arrows, 24 h after tube formation), tube closure at confluence (62 h) and detachment from the alginate wall (red arrows, 91–133.5 h). Colored masks are overlapped on the alginate tube (green) and on the cell monolayer (magenta). 30% Matrigel. Scale bar: 200 µm. (B–D) Middle plane slice (top) and maximum-intensity projections (bottom) of 3D confocal images of the three stages of growth: (B, stage 1) clusters (10% Matrigel, day 7), (C, stage 2) monolayer (30% Matrigel, day 13), (D, stage 3) shrunk (detached) monolayer (30% Matrigel, day 8). Cell membrane, gray (CellMask); inner wall of the alginate tube, dashed green line. Scale bars: 50 µm.

To follow epithelium formation within the alginate tubes at large as well as cellular scales, we took advantage of a lens-free microscope that allows the acquisition of large fields of view (up to 29.4 mm^2^, CMOS sensor) over long periods of time (days) directly in the incubator (Allier et al., 2017). After a holographic reconstruction process, it is possible to obtain a phase image of the biological sample. The resolution is coarse (about 2–3 µm) and there is no sectioning ability but this was sufficient (Hervé et al., 2018) to follow growth of cells within alginate tubes. We could observe the development of the cell monolayer growing under cylindrical constraint over 5.5 days (Fig. 2A; Movie 1).

The growth progression of the tissue can be described as three consecutive stages. Before a monolayer was formed, cells were arranged in clusters of 25–30 µm inside the alginate tube (Fig. 2B, stage 1). These clusters then adhere to the alginate walls forming small monolayer regions that coalesce into a uniformly adherent cell monolayer on the entire inner surface of the alginate tube (Fig. 2C, stage 2). Unexpectedly, upon reaching confluence, contraction of the monolayer led to the formation of a narrower tube than the inner radius of the alginate shell, and caused local or general loss of adhesion to the alginate wall (Fig. 2D, stage 3). In order to investigate the reasons for this cell detachment, we first characterized the proportion of cells at each developmental stage under a range of Matrigel concentrations.

### MDCK cells present higher contractility compared to J3B1A cells

**Fig. 3.**
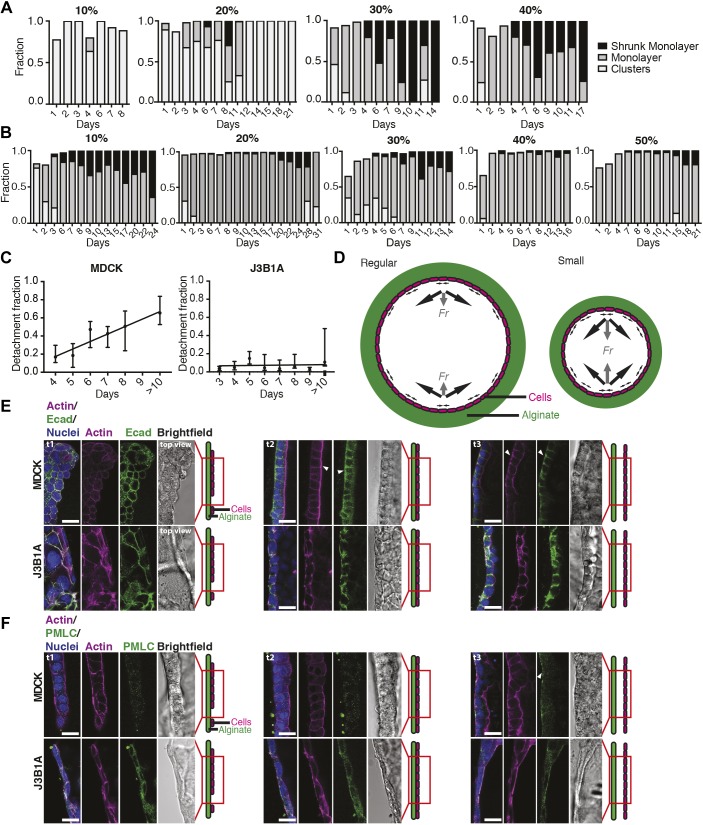
**Comparison of the growth progression for MDCK and J3B1A cells.** (A,B) Histograms of the three stages of MDCK (A) and J3B1A (B) cell growth in alginate tubes with different Matrigel concentrations (10–50%). (C) Mean±s.d. of the detachment fractions for MDCK (left) and J3B1A (right) monolayers over time after tube formation. ‘>10’ denotes 10–13 days. 20–40% Matrigel. *n*=2–26 tubes/day. For MDCK: *Y*=0.0668*X−0.0545, *R*^2^=0.7382, *P*=0.0132 (*, linear regression test, slope compared to zero). For J3B1A: *Y*=0.0036*X−0.0429, *R*^2^=0.06211, *P*=0.5178 (ns). (D) Diagram of regular (left) and small (right) tube orthogonal sections. Cells (magenta) forming a monolayer on the alginate wall (green) produce contractile forces (black arrows) with a bigger resulting force (*Fr*, gray arrows) in the small tubes. (E,F) Confocal microscopy images of F-actin with E-cadherin (Ecad) (E) or PMLC (F) in MDCK (top) and J3B1A (bottom) cells along the monolayer growth (t1, stage 1, separated groups of cells; t2, stage 2, cell monolayer; t3, stage 3, detached monolayer). 30% Matrigel. White arrowheads indicate protein accumulations in the tissue. Scale bars: 20 µm.

Cell shape can change through cell contractility, which strongly depends on ECM adhesion. Together with cell–cell junctions and the actomyosin cortex, which transmits mechanical forces to the other cells of the tissue, the cortex of the basal side contributes to effective epithelial tension (Bielmeier et al., 2016). Thus, modulating the concentration of Matrigel may significantly influence tissue formation and behavior. We imaged alginate tubes containing MDCK and J3B1A cells using bright-field imaging for days after encapsulation, and for several Matrigel concentrations as a percentage of the total volume of the CS solution (Fig. 1A). At 10% concentration, the Matrigel did not homogeneously coat the entire inner surface of the alginate tubes, as observed using confocal imaging (Fig. S1A) and as previously published (Alessandri et al., 2016). At this concentration, MDCK cells could not form a proper cell monolayer, and they predominantly remained as clusters (Fig. 3A). However, J3B1A cells, though with slower dynamics than for higher concentrations of Matrigel, spread onto the entire surface of the alginate tubes (Fig. 3B). In this case, the cell monolayer rapidly detached. This suggests that the holes present in the Matrigel coat below 20% concentration do not prevent J3B1A cells from forming a monolayer, but do not provide sufficient adhesion to the alginate substrate. By contrast, at Matrigel concentrations above 40%, J3B1A cells formed spheroidal cysts (data not shown), probably because Matrigel was present in the lumen of the alginate tube at this concentration (Fig. S1A).

In order to further explore the implication of adhesion in the monolayer detachment process, we observed Matrigel distribution in tubes with and without cells and we selected the intermediate concentration of 30% (Fig. S1B–D). Interestingly, we observed: 1) that Matrigel was homogeneous and stable over time in tubes without cells, 2) that MDCK cells occasionally remodeled the Matrigel distribution on the basal side of the tissue but that Matrigel coating was still present on the alginate surface after monolayer detachment, and 3) that J3B1A cells did not change Matrigel distribution during their growth. Taken together, these results support the conclusion that adhesion loss is not the origin of cell detachment. However, the capability of MDCK cells to rearrange the adhesive matrix may enhance this phenomenon.

It has been recently shown that dividing cells exhibit lower tension at intercellular contacts and lower protein levels of E-cadherin at cell junctions (Miroshnikova et al., 2018). Thus, different dividing rates in MDCK and J3B1A cells could account for their different abilities to detach. We therefore inhibited cell proliferation through mitomycin-C treatment (Fig. S2). We observed that after blocking cell division, monolayer detachment increased, probably owing to a more efficient and homogeneous contractility into the tissue. This implies that the detachment process is not driven by cell proliferation.

These results targeted a role of cell contractility in the detachment process. We investigated whether cell contractility changed during the formation of the cell monolayer. For concentrations of Matrigel between 20% and 40%, both MDCK and J3B1A cells formed a monolayer within 3–4 days post-encapsulation, and this monolayer detached rapidly for MDCK cells (4–6 days) (Fig. 3A, ‘shrunk monolayer’), while detachment was more limited for J3B1A cells (Fig. 3B). For MDCK cells, the detachment fraction linearly increased over time, indicating a progressive contraction of the tissue once it has formed a complete monolayer (after 3–4 days). The detachment of the monolayer was therefore time-dependent in MDCK cells, unlike J3B1A cells, which showed a slight and almost constant detachment fraction after the monolayer formation (Fig. 3C). We propose that this may be explained by the stronger contractile forces that MDCK tissues can generate, as compared to J3B1A cells. In order to confirm our hypothesis, we analyzed cell–cell adhesion complexes and cell actomyosin organization in both cell lines at different stages of growth in the alginate tubes (Fig. 3E,F). Using immunofluorescence of F-actin, E-cadherin and phosphorylated myosin light chain II (p-MYL2, PMLC henceforth), we first observed that MDCK cells gradually polarized with cell monolayer formation, as observed from the basolateral localization of E-cadherin and apical localization of actin (Fig. 3E, t2, white arrowheads). Upon detachment, strikingly, E-cadherin relocalized to the basal membrane (Fig. 3E, t3, white arrowheads). Moreover, phosphorylated myosin and actin, essential players in cell contractility, relocated to the basal side of detached monolayers (Fig. 3E,F, t3, white arrowheads), supporting the notion that MDCK cells become more contractile on their basal side concomitantly with detachment. In comparison, J3B1A cells showed more stress fibers but less organized E-cadherin and PMLC staining (Fig. 3F). These results highlight that J3B1A cells are less polarized in the tissue and are more squamous than MDCK cells. J3B1A cells also did not show the striking relocalization of actin and PLMC in detached monolayers. Taken together, our results support the notion that MDCK cells have higher contractility, linked to a well-organized actomyosin system that relocalizes to the basal pole upon detachment.

In order to confirm the main role played by cell contractility in detachment, we disrupted myosin activity using blebbistatin, a drug known to strongly reduce the active tension of MDCK monolayers (Vincent et al., 2015). Under conditions of blebbistatin treatment, monolayer detachment did not occur, even after few days of treatment (Fig. S2). This result clearly shows that cell contractility is the driving force of detachment.

In the tubular geometry, contractile forces within the plane of the cell monolayer result in radial forces perpendicular to the tube surface that pull the cells inward. One might expect that the intensity of these radial forces is directly linked to the curvature of the tube; a smaller radius results in a larger radial force, if the contractile forces stay the same (Fig. 3D). Consistent with this reasoning, we observed both a faster appearance of shrunk J3B1A monolayers and an increase of the detachment fraction over time in the smaller alginate tubes (Fig. S3A; Fig. 5C). Using the same reasoning, one might expect that in curved tubes, detachment would be faster and more pronounced in the outer side of the curve. Thus, we went on to study the detachment dynamics of cell monolayers in curved tubes.

### Monolayer detachment is favored on the outer side of curved tubes

**Fig. 4.**
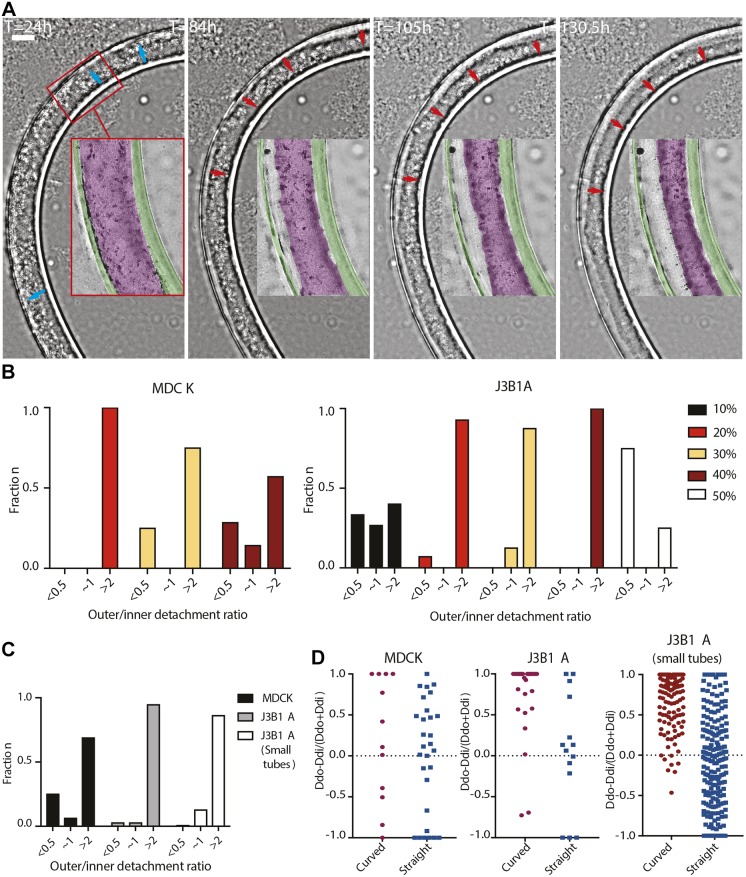
**Epithelia detachment in curved tubes.** (A) Frames of J3B1A cell monolayer growth in a curved tube. Live imaging performed across a 5.5 day range with a lens-free microscopy (phase images). Insets show monolayer progression (blue arrows, 24 h after tube formation) and the progressive detachment from the outer part of the alginate wall (red arrows, 84–130.5 h). Colored masks are overlapped on the alginate tube (green) and on the cell monolayer (magenta). 30% Matrigel. Scale bar: 200 µm. (B) Histograms of the detachment fraction between the outer and the inner side of the curved tube, for MDCK (left) and J3B1A (right) cells at different Matrigel concentrations (10–50%). ‘∼1’ denotes ratio of 0.5–2. (C) Histograms of the detachment fraction of MDCK (black) and J3B1A (gray) monolayers in regular tubes and J3B1A (white) monolayers in small tubes. (D) Ratios of detachment on the outer compared to the inner side of the tube bend for both cell lines. *Dd*, detachment fraction; *i*, inner side of the bend (red circles), right side of the straight tubes (blue squares); *o*, outer side of the bend, left side of the straight tubes. (B–D) *n*=1–7 MDCK curved tubes/condition, *n*=7–15 J3B1A curved tubes/condition in B; *n*=12 MDCK curved tubes, *n*=30 J3B1A curved tubes, *n*=121 J3B1A small curved tubes in C and D; *n*=29 MDCK straight tubes, *n*=14 J3B1A straight tubes, *n*=196 J3B1A small straight tubes in D. Data obtained from tubes after day 4 and with a detachment fraction >5% (20–40% Matrigel).

As tubes were formed and packed in petri dishes for cell culture, they adopted curved configurations. Long-term live-imaging of a tube of J3B1A cells at a bend of the tube showed the dynamics of the detachment process (Fig. 4A; Movie 2). 84 h after the tube formation, shrinkage of the monolayer clearly appeared at the outer part of the bend. This continued until the total detachment of the monolayer from the alginate wall on the outer side of the curve (130 h). To quantitatively compare the frequency of detachment between the inner and outer side of a bend, we therefore took into account tubes with shrunk monolayers or cell extrusion from 4 days after formation, i.e. once cells reached confluence (Fig. 3A,B). By taking the ratio of the outer and inner detachment fraction (using 10^−5^ as 0 for no detachment of the inner side), we can distinguish three groups of values (Fig. S3C): under 0.01 (i.e. almost exclusively detachment on the inner side of the bend), over 100 (i.e. almost exclusively detachment on the outer side of the bend), and around 1 (similar detachment fractions on both sides). However, this latter population was highly displaced towards values above 1. Thus, we considered asymmetric detachment for ratio values above 2 or below 0.5, meaning that the cell monolayer on one side detaches with a detachment length at least two times bigger than the other side of the tube. This accounts for the asymmetry observed in ratio values closer to 1. Moreover, we only considered tubes with at least 5% detachment to avoid including detachment linked to local heterogeneities of the substrate (Fig. S3B). A homogeneous and stable Matrigel distribution over time was also observed in the case of curved tubes (Fig. S1E), excluding asymmetric adhesive coating as a potential cause of asymmetric monolayer detachment. The asymmetry in monolayer detachment on the outer side of the curved tube was the greatest at a 20% Matrigel concentration for MDCK cells, whereas for J3B1A cells, the peak occurred at 40%. However, in J3B1A cells, asymmetric detachment was lower at 10% Matrigel and inverted at 50% Matrigel (Fig. 4B).

**Fig. 5.**
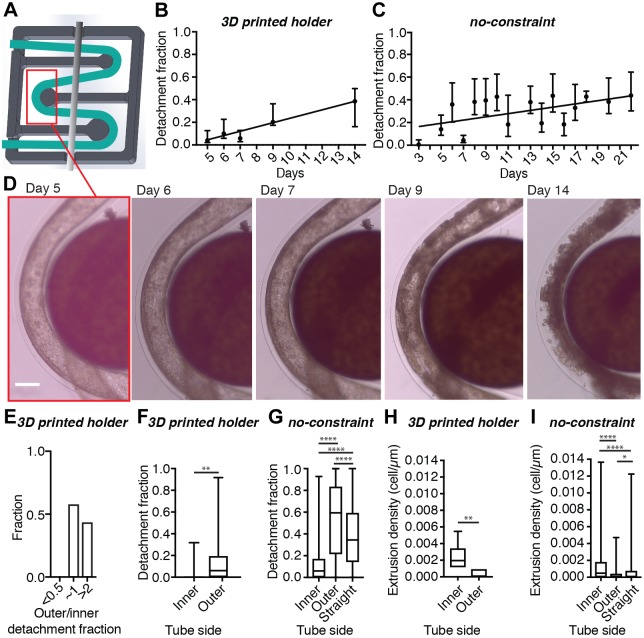
**Epithelial monolayer growth in curved small tubes under controlled curved constraint (30% Matrigel).** (A) Design of the 3D printed tube holder (dark gray, 14×14 mm^2^), where the position of the alginate tube (green) is maintained by a metal bar (light gray). (B,C) Mean±s.d. of the detachment fractions, on both sides of the tube, of J3B1A monolayers over time after tube formation. (B) Small curved tubes in the holder. *Y*=0.0376*X−0.1442, *R*^2^=0.9179, *P*=0.0102 (*, linear regression test, slope compared to zero). *n*=3–6 tubes/day. (C) Small curved tubes without any constraint. *Y*=0.0102*X–0.1825, *R*^2^=0.1729, *P*=0.1232 (ns, linear regression test, slope compared to zero). *n*=2–33 tubes/day. 20–40% Matrigel. (D) Bright-field images of the progression of J3B1A cell growth around a pillar of the tube holder. Scale bar: 200 µm. (E) Histograms of the detachment fraction between the outer and the inner side of the curved tube of J3B1A monolayers. Data obtained (*n*=15) from tubes after day 4 and with a detachment fraction>5%. (F,G) Detachment fraction on the inner and the outer side of curved tubes with constraint (F, **P=0.0024 inner versus outer) and without constraint (G, ****P<0.0001 inner versus outer), compared to straight tubes (G, ****P<0.0001 straight versus inner and straight versus outer). (H,I) Cell extrusion densities on the inner and the outer side of the curved tube with holder (H, **P=0.0022 inner versus outer) and without constraint (I, ****P<0.0001 inner versus outer) compared to straight tubes (I, ****P<0.0001 straight versus inner, *P=0.0490 straight versus outer). All statistical comparisons by Mann–Whitney test. (F–I) Top and bottom of box indicate 75th and 25th quartiles, respectively; whiskers indicate the min and the max values; the middle line is the median. F and H, *n*=23 curved tubes; G, *n*=119 curved tubes; I, *n*=139 curved tubes; G and I, *n*=403 straight tubes. 20–40% Matrigel. Data obtained from tubes after day 4.

Interestingly, when results from 20% to 40% Matrigel concentrations are combined, although J3B1A cells globally detached less than MDCK cells (Fig. 3C), they presented a higher asymmetric detachment in curved tubes. Furthermore, for J3B1A cells, the total detachment fraction was higher in small tubes (Fig. 5C) than in regular tubes (Fig. 3C) but the asymmetric detachment fraction was lower (Fig. 4C,D). This confirms that the pulling force resulting from cell monolayer contraction is higher in small tubes and suggests that asymmetric detachment is dependent on the overall contractile force of the cell monolayer. Above a certain threshold of contractility, the monolayer detaches homogeneously, while below the threshold, detachment is strongly favored in the outer side of the curved tubes. Although our results show that contractile forces combine with the curvature of the substrate to control detachment, the high variability of the tube curvature made us look for a more controlled system of tube shape.

### Controlled curvature of the tube bend enhances the mechanical response of J3B1A cells

Since curved tubes promoted asymmetric monolayer detachment on the outer side of the bend, we evaluated quantitatively how curvature controls detachment by plotting the detachment fraction as a function of the curvature radius of the bends (Fig. S3D). Although we observed that the detachment fraction for MDCK cells appeared to increase for higher curvatures in regular tubes, the overall detachment fraction did not significantly depend on the curvature of the tube bend. Similar results were obtained in small tubes with J3B1A cells (Fig. S3E). Since none of these results were statistically significant, we looked for a way to obtain more controlled values of curvature.

We designed a tube holder (Fig. 5A,D) in which tubes could be curved around cylindrical pillars of a given radius, fixing the curvature of the bends. Using this device, we were able to obtain curvature radii as small as one millimeter. We then followed the tissue development of J3B1A cells in small tubes fixed in this controlled configuration with defined radius of curvature (Fig. 5B). This holder also represents an alternative to using agarose to immobilize the tubes, with the advantage of limiting any barriers to diffusion of gases and nutrients around the cells. Using this holder, we observed a more regular increase of J3B1A monolayer detachment over time (Fig. 5B) than in small tubes without any constraints (Fig. 5C), confirming that higher curvature can compensate for the weak contractility of J3B1A cells to promote detachment. Consistent with our previous observations, the asymmetry of detachment was reduced for higher detachment rates (Fig. 5E). Moreover, the ability to more accurately control the bending of the tubes confirms the previous results (Fig. 5F,G).

During these experiments, we consistently observed extrusion of cells on the basal side of the epithelium towards the inner side of the curve in the tube, with a significantly higher density compared to straight tubes (Fig. 5H,I). In an epithelium, cell proliferation and cell removal must be balanced in order to keep the tissue surface and shape constant. Local contraction of cells promotes extrusion out of the epithelium (Kocgozlu et al., 2016; Wu et al., 2015). Since detachment rate is also driven by contraction and can be enhanced by curvature, it is possible that extrusions, promoted by constriction of the epithelial cell actomyosin ring, could be affected by the same mechanism.

### Cell extrusion predominantly occurs in the inner side of curved tubes

**Fig. 6.**
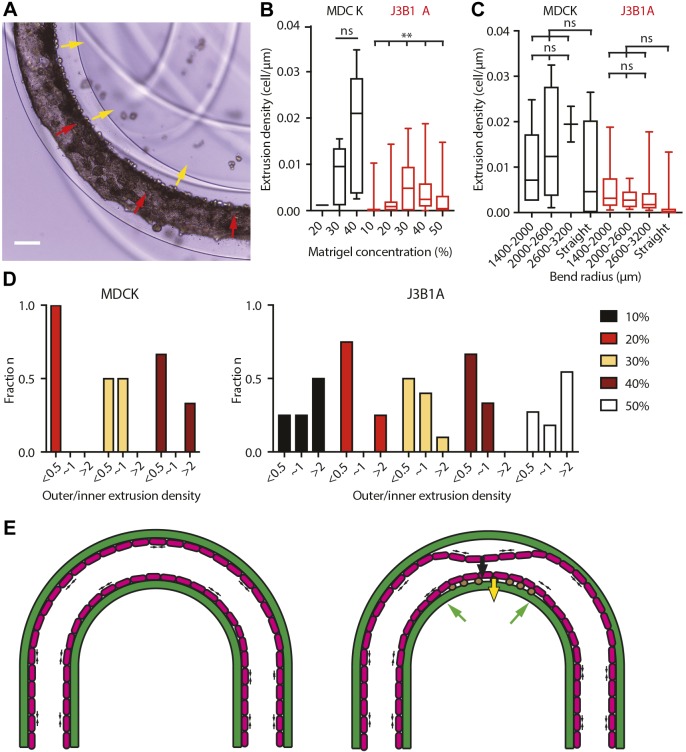
**Cell extrusion in curved tubes.** (A) Bright-field image of MDCK tissue in a curved tube showing its detachment on the outer side of the bend (red arrows) and the cell extrusions (yellow arrows) on the inner side (40% Matrigel, day 7). Scale bar: 200 µm. (B,C) Cell extrusion densities on the inner side of the curved tube for both cell lines (MDCK, black; J3B1A, red). (B) Cell extrusion densities at Matrigel concentrations of 10–50%. MDCK: *P*=0.4376 (ns) across 30–40% by Mann–Whitney test. J3B1A: **P=0.0097 across 10–50% by Kruskal–Wallis test. (C) Cell extrusion densities at different curvature radii of the tube bend [MDCK: *P*=0.4417 (ns); J3B1A: *P*=0.3757 (ns) by Kruskal–Wallis test] and comparing curved to straight tubes [MDCK: *P*=0.7636 (ns); J3B1A: *P*=0.5432 (ns) by Mann–Whitney test]. Top and bottom of a box indicate 75th and 25th quartiles, respectively; whiskers indicate the min and the max values; the middle line is the median. B, *n*=1–9 MDCK tubes/condition, *n*=15–45 J3B1A tubes/condition; C, *n*=2–32 MDCK tubes/condition, *n*=8–51 J3B1A tubes/condition. Data obtained from tubes after day 4. (D) Histograms of extrusion density fractions between the outer and the inner side of the curved tube, for both cell lines (MDCK, left; J3B1A, right) at different Matrigel concentrations (10–50%). ‘∼1’ denotes ratio of 0.5–2. *n*=1–9 MDCK tubes/condition, *n*=4–16 J3B1A tubes/condition. Data was obtained from tubes after day 4 and at a cell extrusion density >0.002 cell µm^−1^. (E) Diagram of the tube section showing the contractile lateral forces (small black arrows) produced by the cell monolayer (magenta) formation in a curved alginate tube (green). On the right, the resulting forces induce monolayer detachment from the outer side of the bend (large black arrow) and cell extrusion (brown dots) on the inner side (yellow arrow), where the pulling force is balanced by the alginate wall (green arrows).

We systematically quantified extrusion densities to fully characterize behaviors of both cell lines in straight and curved tubes. With the same approach used for monolayer detachment, we only considered tubes with an extrusion density of at least 0.002 cells µm^−1^ to avoid including extrusions linked to local heterogeneities of the substrate (Fig. S3B). Moreover, we considered asymmetric cell density between the two sides of the curved tubes for ratio values above 2 or below 0.5, meaning that the cell extrusion density on one side is at least two times bigger than on the other side of the tube. Once at confluence, intercellular forces into the monolayer created compressive patterns and extruded some cells, eventually forming clusters on its surface. Surprisingly, in the alginate tubes, extrusion upon confluence (after 100 h) occurred towards the Matrigel layer on the basal side of the monolayer (Fig. 6A). MDCK cells showed a mean extruded cell density, expressed as the number of extruded cells per μm, several times higher than J3B1A cells (Fig. 6B,C). Consistent with the higher detachment fraction of MDCK than of J3B1A cells, this result reinforces the hypothesis of a higher contractility of MDCK than J3B1A cells. As observed for detachment, curved tubes appeared to have a higher extrusion density, but it was not significantly dependent on the exact curvature of the bend. Consistent with detachment observations, J3B1A cells were more asymmetrically extruded than MDCK cells (Fig. 6D). Overall, extrusion probability mirrors monolayer detachment probability (Fig. 4B). Indeed, we found for both cell lines that extrusion predominantly occurred on the inner side of the bends. However, this asymmetry is reversed at 10% and 50% Matrigel concentrations. The lack and/or excess of adhesive molecules at high and low Matrigel concentrations is probably the cause of this behavior. Indeed, the heterogeneous distribution of the Matrigel observed at 10% (Fig. S1A) could reduce the cell adhesion, leading to lower intracellular lateral forces and therefore to a lower cell extrusion. In the opposite case, the thicker Matrigel layer when the concentration was 50% (Fig. S1A) could firstly reduce the constraints on extrusion of cells toward the alginate wall and secondly enhance contractility of cells through the stronger attachment of the monolayer to its ECM, leading to a less asymmetric extrusion. As shown in Fig. 5H,I, in small alginate tubes with J3B1A cells, the difference in extruded cell density between the inner and outer of a curved tube was significant. It confirmed the role that curvature has on tissue cell reorganization, as inducing an asymmetric geometry results in an asymmetric extrusion density.

These results, mirroring the ones obtained for monolayer detachment, support that cell contractility is also the cause of extrusion. The asymmetry observed in curved tubes (detachment of the monolayer on the outer side, extrusion on the inner side) may arise from the direction of the forces resulting from contraction: while the resulting force of contraction is pulling the monolayer off the alginate shell, leading to detachment on the outer side, the resulting force is pushing the cells towards the alginate shell on the inner side, leading to cell extrusion (Fig. 6E).

## DISCUSSION

Here, we show that epithelial cells growing under tubular confinement may develop into cellular tissues of different sizes and shapes, resulting from a combination of cellular properties and the initial shape of the cell confiner. By growing cells into hollow tubes coated with Matrigel, we observed that cells form a complete monolayer after a few days. However, while the epithelium layer of MDCK cells constricted soon after confluence and detached from the tubular substrate to finally form a narrow tube, J3B1A cells essentially remained attached to the substrate. Since these differences depended on self-imposed forces within the tissue, we concluded that the final size and shape also depended on cell contractility and on curvature. Excluding the role of ECM remodeling in detachment, the observations that MDCK monolayers have a more regular and ordered structure when compared to J3B1A monolayers and that the actomyosin machinery relocalized to the basal membrane in MDCK cells support the notion that MDCK cells have a larger contractility than J3B1A. Moreover, cell extrusion on the basal side of MDCK monolayers was more pronounced than for J3B1A cells, also supporting that the MDCK basal side is more contractile. However, detachment was enhanced in thinner tubes (smaller diameter) or well-controlled curved tubes of J3B1A cells. These results suggest that the forces for detachment result from cell contractility integrated over the curved surface, and that higher curvature can compensate for weaker contractile forces in order to provide the same resulting detachment forces. Blocking myosin activity, and therefore contractility, inhibited monolayer detachment. Blocking cell proliferation, thereby increasing contractility, increases monolayer detachment. These observations reinforce the hypothesis of a collective effect of tension upon confluence.

Moreover, by bending the tubes, which corresponds to curvature other than the tube radius, we observed an asymmetry of cell detachment, with cell layers detaching mainly from the outer side of curves. This asymmetry was mirrored by a significant proportion of cells extruded in the inner side of bends in the tubes. J3B1A monolayers displayed these drastic shape changes upon reaching confluence in a more pronounced manner than MDCK cells. This is probably due to the lower contractility of J3B1A cells leading to a lower detachment fraction and extrusion density. We proposed a model of resulting forces in curved tubes (Fig. 6E) that highlights a subtle coupling between contractile forces and curvature that is partially masked when the cell contractility is higher, as for MDCK cells.

Matrigel concentration also affects the microenvironment and, because adhesion is essential for proper epithelial growth, the characteristics and development of the tissue. In both cell lines, Matrigel concentrations in the range of 20–40% increased the asymmetry of detachment and extrusion. At 10% Matrigel, there was no difference in detachment of J3B1A monolayers between the inner and outer parts of the tube bend, and MDCK did not reach monolayer formation. Indeed, if low Matrigel concentrations confer a weaker attachment of the cell monolayer to its substrate, contractile forces are expected to be insufficient to detach the monolayer. By contrast, at 50% Matrigel, the Matrigel layer was so thick that it did not provide the cells with a smooth surface to adhere to. This probably masks the effect of curvature, as the epithelium layer does not follow the curvature of the tube.

Taken together, we show here that epithelial tissue can grow under cylindrical confinement. We show that the shape, size and adhesion properties of the substrate significantly affect the final morphology of the epithelium and ongoing cell reorganization (monolayer detachment, extrusion). Thus, our findings provide instrumental information for the bioengineering field focusing on the control of tissue growth *in vitro*.

## MATERIALS AND METHODS

### 3D printing and assembling of the microfluidic device and tube holder

The microfluidic device was printed with a design of three co-axial channels as described in Alessandri et al. (2016). An EnvisionTEC 3D printer was used under conventional working conditions. Prints were made with HTM 140 V2 resin supplied by EnvisionTEC (Gladbeck, Germany), which provides a resolution in the *z* axis of 25 µm. The process typically takes around 3 h. The chip was extensively and gently rinsed with ethanol in order to remove the liquid resin from the inner canals. It was then sonicated for 20 s and exposed to UV light for 15 min. Three 19-gauge stainless steel segments of 2–3 cm with smoothed extremities were glued with epoxy resin (Loctite M-31CL; RS Components, Corby, UK) at the entry of each channel. Polydimethylsiloxane (PDMS; Dow Corning, Midland, MI) was spread on the side of the hole of the chip tip to reduce surface tension. This treatment was crucial to get a regular jet at the exit of the device. Devices with holes of two different inner diameters were printed, one of 200 μm (‘regular device’) and one of 120 μm (‘small device’). The small device had the smallest diameter for which the 3D printer resolution allowed the printing of functional channels.

The tube holder was printed with dimensions of 14 mm×14 mm in order to fit perfectly across the 20 mm diameter of a glass-bottom dish (P50G-1.5-30-F; MatTek, Bratislava, Slovakia). Two snicks were present on the lateral side in order to fit a stainless-steel tube (19G) that prevented the alginate tube from floating in the medium. We designed the 3D printed holder to have cylindrical pillars of different radii of curvature (0.8 mm, 1.0 mm, 1.2 mm, 1.4 mm and 1.6 mm).

### Solutions and cell suspension preparation

Alginate was prepared by dissolving 2.5% w/v sodium alginate (Protanal LF200S; FMC corporation, Philadelphia, USA) in water and adding 0.5 mM of sodium dodecyl sulfate surfactant (SDS; A394; ITW Reagents, Darmstadt, Germany). The solution was filtered with a 1 μm glass filter (Acrodisc syringe filter, 4523; Pall Life Sciences, New York, USA). The alginate solution was labeled with Alexa Fluor 488–TFP (A30005; Invitrogen, Thermo Fisher Scientific, Waltham, USA) at 100 mg l^−1^, then subjected to EDC-mediated coupling with sulfo-NHS. It was finally dialyzed with a Slide-A-Lyzer cassette 10K 12–20 ml capacity (Thermo Fisher Scientific, Waltham, USA) in water for 24 h, with the water changed every few hours (∼4 h during the daytime). The intermediate solution (IS) was sorbitol (56755; Merck, Darmstadt, Germany) dissolved in milliQ water at a concentration of 300 mM and filtered using a Steritop-GV PVDF filter with 0.22 µm pores (MPSCGVT05RE; Merck). The cell solution (CS) at the innermost canal of the chip was prepared from a filtered suspension of cells (40 μm filter with a nylon cell strainer, 431750; Corning, Durham, USA) at a concentration of ∼3×10^7^ cells ml^−1^ in a total volume of 300 µl of sorbitol and Matrigel (354234, Lot #5173011; Corning). The solution was prepared with different Matrigel concentrations from 10% to 50% v/v. For adhesive protein (Matrigel) visualization, red fluorescent rhodamine-labeled laminin (LMN01-A; Cytoskeleton Inc., Denver, USA) was added to the Matrigel solution at a concentration of 10% v/v.

### Cell culture

EpH4-J3B1A mammary epithelial cells (J3B1A) and Madin–Darby canine kidney cells (MDCK) were used. The cell growth medium for MDCK cells was high glucose Dulbecco's Modified Eagle’s Medium (DMEM; 61965026; Thermo Fisher Scientific) supplemented with 10% fetal bovine serum (FBS, 102701036; Thermo Fisher Scientific), 1% penicillin-streptomycin (15140122, Thermo Fisher Scientific) and 1% non-essential amino acids solution (NEAA, 11140035; Thermo Fisher Scientific). The J3B1A growth medium was low glucose Dulbecco's Modified Eagle’s Medium (DMEM, 21885025; Thermo Fisher Scientific) supplemented with 10% HI Bovine serum (26170043; Thermo Fisher Scientific) and 1% penicillin-streptomycin.

Cell detachment from the culture flask has a different protocol for each cell line. For MDCK cells, cells were incubated in 0.05% trypsin-EDTA (25300054; Thermo Fisher Scientific) for 10 min at 37°C. For J3B1A cells, cells were incubated in Versene (15040033; Thermo Fisher Scientific) for 30 min at 37°C, and then 0.05% trypsin-EDTA was added and the cells incubated at 37°C for a further 5 min.

Both cell lines were regularly tested negative for contamination with mycoplasma (Eurofins GATC Biotech, Konstanz, Germany).

### Tube formation

Three pump-connected syringes (10MDR-LL-GT SGE; Analytical Science, Victoria, Australia) contained the solutions (alginate for the external channel and sorbitol for the intermediate and the inner channels) employed for tube formation, as described above. The alginate flow rate was set at 115 ml h^−1^ for the regular device, and at 65 ml h^−1^ for the small device. The sorbitol flow rate was set at 105 ml h^−1^ for the regular device, and at 55 ml h^−1^ for the small device. The flow rate of the cell solution syringe was set at 90 ml h^−1^ for the regular device, and at 48 ml h^−1^ for the small device.

The alginate and the sorbitol solutions were loaded directly from the syringes to the connections on the device. The tube containing the cell solution was maintained in ice in order to keep the Matrigel under its gelation point (4°C). It was then plugged with the pump-connected syringe of sorbitol.

The gelation bath with 0.1 M calcium chloride (449709; Sigma-Aldrich, St Louis, USA) was pre-warmed to 37°C.

The tip of the device was dried before each tube production to avoid capillarity effects. Once the three pumps were simultaneously set off, the tip of the device was immersed in the calcium bath and a tube of ∼1 m in length could nominally be produced while the cell solution was flowing out. Afterward, calcium chloride was eliminated through aspiration with a sterile needle. The tube was washed with medium without serum in order to avoid aggregates of precipitated proteins, and the wash medium then replaced by cell growth culture medium. The tubes were kept in an incubator with controlled environment (37°C, 5% CO_2_). Medium was changed every 2–3 days.

Between each experiment, the chip was extensively cleaned with deionized water, ethanol, deionized water, phosphate-buffered saline (PBS; 18912014; Thermo Fisher Scientific) and deionized water again.

### Immunofluorescence staining

Tubes were fixed in 4% formaldehyde solution (F8775; Sigma-Aldrich) in phenol red-free minimum essential medium (MEM; 51200046; Thermo Fisher Scientific) for 20 min at room temperature, washed twice with phenol red-free MEM and stored at 4°C. After cell fixation, cells were stained with CellMask Deep Red Plasma Membrane Dye (1:1000; C10046; Molecular Probes, Thermo Fisher Scientific) for 60 min at room temperature or with Hoechst 33342 (1:1000; 62249; Thermo Fisher Scientific) for 10 min at room temperature. For immunostaining, alginate tubes were first dissolved in PBS for 2 h. Cells were then permeabilized using 0.1% saponin (A4518,0100; Axonlab, Le Mont-sur-Lausanne, Switzerland) in PBS and blocked in 1% gelatin PBS solution (G7765; Sigma-Aldrich) for 45 min. Primary antibodies were incubated for 1 h in 1% gelatin buffer. Mouse anti-E-cadherin (610181; BD Biosciences, Allschwill, Switzerland) and rabbit anti-phospho-myosin light chain 2 (3671; Cell Signaling Technology, Danvers, USA) antibodies were used at 1:50 dilution in PBS. F-actin was stained with SiR-Actin (1 µM; SC-001; Spirochrome, Stein am Rhein, Switzerland) incubated for 45 min with the secondary antibodies diluted at 1:250 in PBS (donkey anti-mouse Alexa Fluor 488, A21202; donkey anti-rabbit Alexa Fluor 488, A21206; Invitrogen, Thermo Fisher Scientific) in 1% gelatin buffer protected from light. Samples were washed three times with PBS after each antibody or fluorescent probe incubation.

### Confocal imaging

Confocal images were obtained using Nikon Eclipse Ti microscope with a Zyla sCMOS camera with either an Apo LWD 40×/1.15 water immersion objective (Nikon) or a plan 20x/0.45 objective. Tubes were maintained in a glass-bottom dish with 5 mg ml^−1^ low melting temperature agarose (A9414; Sigma-Aldrich) covered by phenol red-free MEM. An upright LSM 710 microscope with a Plan-Apochromat 20x/1.0 DIC objective (Carl Zeiss) was used to get confocal images of the tubes with green-fluorescent alginate and red-fluorescent laminin. Confocal images of the immunostained samples were obtained using a LSM 780 microscope with a C-Apochromat 40×/1.2 water immersion objective (Carl Zeiss) directly in a glass-bottom dish.

### Live imaging

Live imaging was performed with a Cytonote lens-free video microscope (Iprasense). It is based on the lens-free imaging system described by Su et al. (2009), which was modified to perform continuous monitoring inside an incubator at a controlled temperature and humidity. It uses a CMOS image sensor with a pixel pitch of 1.67 µm and an imaging area of 6.4×4.6=29.4 mm^2^. Multiple wavelength illumination is provided using multichip LEDs delivering red, green and blue illuminations. The RGB LEDs are located above a 150 µm pinhole, at a distance of ∼5 cm from the cells. The phase image of the sample is obtained through a multispectral TV optimizer (Hervé et al., 2018). Live imaging was performed during 112 h in an incubator with controlled environment (37°C, 5% CO_2_). Tubes were maintained in a glass-bottom dish with 6 mg ml^−1^ low melting temperature agarose covered by cell growth medium with phenol red-free MEM.

### Drug treatments

Samples were incubated with drugs at day 3 for MDCK cells and day 6 for J3B1A cells (immediately after the cell monolayer formation) for observations over time. To maximize inhibition of cell proliferation and minimize drug toxicity, cells were incubated for 1 h with 15 µM mitomycin-C (M4287; Sigma-Aldrich), then washed out with warm medium, and 6 h later, incubated for a further 1 h with 15 µm mitomycin-C. To inhibit myosin activity, 20 µM blebbistatin (B0560; Sigma-Aldrich) in DMSO was added and kept in the growth media until day 7 (MDCK cells) or day 9 (J3B1A cells). Cells were incubated in DMSO for the same amount of time as control.

### Data collection and analysis

Data on tissue states, i.e. monolayer detachment, clusters and cell extrusions, were obtained from bright-field images taken each day throughout progression of tissue growth. At least two images were taken for each tube, with a 5× PHO objective on a Leica S 40/0.45 digital camera. Data from alginate tubes prepared under the same conditions (i.e. microfluidic device, Matrigel concentration and cell line) but at different days were collected together. Image analysis was performed with ImageJ.

Fraction of detachment was calculated as the ratio of the length the alginate wall with an absence of monolayer due to its detachment (*Dd*) versus the overall length of the tube segment (*Dt*). We evaluated the difference in length of the detached monolayer of the tube at the inner (*Ddi*) and the outer (*Ddo*) part of the curved tube divided by the total length of both the inner and outer tube segment (*Dti* and *Dto*). We compared these detachment fractions between both sides of the curved tube with the following equation: 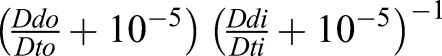, where 10^−5^ was arbitrarily added to avoid infinite values in case of no detachment on the inner side.

Unless otherwise stated in the figure legend, data and images presented were obtained with regular size tubes and data are expressed as mean±s.d. All sample numbers and the *P*-values are reported in the figure legends. *P*>0.05 was considered as not significant (ns). Each experiment was repeated at least three times. All statistical tests were performed using Prism software (GraphPad). Since data distributions did not follow normality assumptions, non-parametric tests were used (Mann–Whitney and Kruskal–Wallis). When the highly spread and asymmetric distributions of the data were not adapted to apply standard statistical tests, direct representation of the distributions was chosen for a greater readability of the results.

## Supplementary Material

Supplementary information
