# Supplementary material for: Curvature-dependent constraints drive remodeling of epithelia
Source: J Cell Sci. 2019 Jan 24;132(4):jcs222372. doi: 10.1242/jcs.222372 (PMC6398478; doi:10.1242/jcs.222372)
Supplement: Supplementary information [file joces-132-222372-s1.pdf]

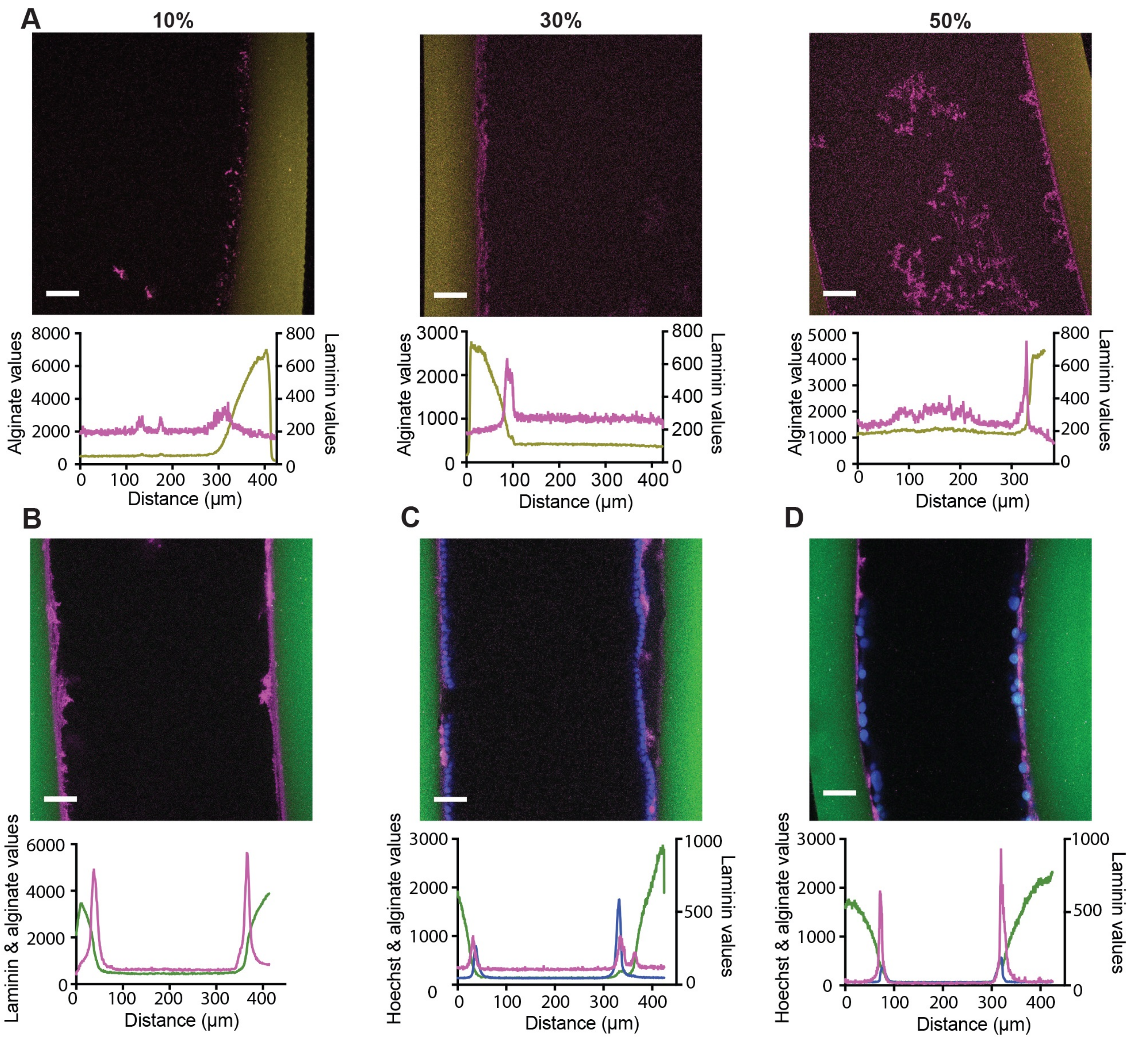

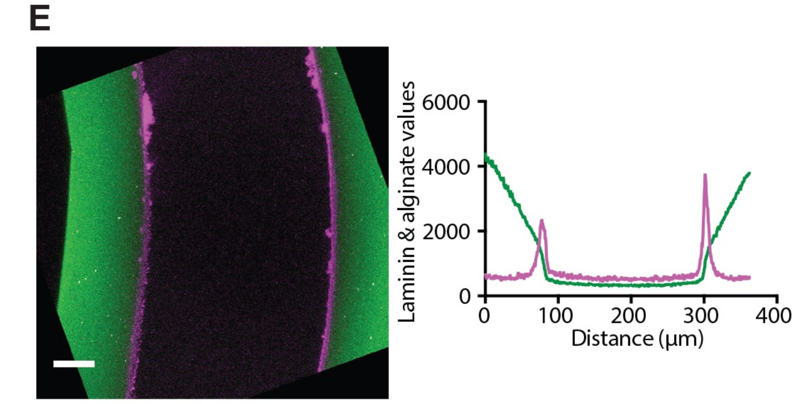

Figure S1. (A) Top: confocal images of the equatorial plane of representative alginate (yellow) tubes formed with 10%, 30% and 50% of Matrigel concentrations (magenta). Scale bars: 100 µm. Bottom: respective average signal intensities along the tube section, of the alginate and of the laminin. (B-E) Top: confocal images of the equatorial plane of representative alginate (green) tubes formed with 30% of Matrigel concentration (magenta) after 3 days in culture conditions (cell medium, 37°C, 5% CO2). (B,E): without cells, straight (B) and curved (E) tubes. (C,D): straight tubes with MDCK (C) and J3B1A (D) cells (blue, nuclei, Hoechst). Scale bars: 100 µm. Bottom or right: respective average signal intensities along the tube section.

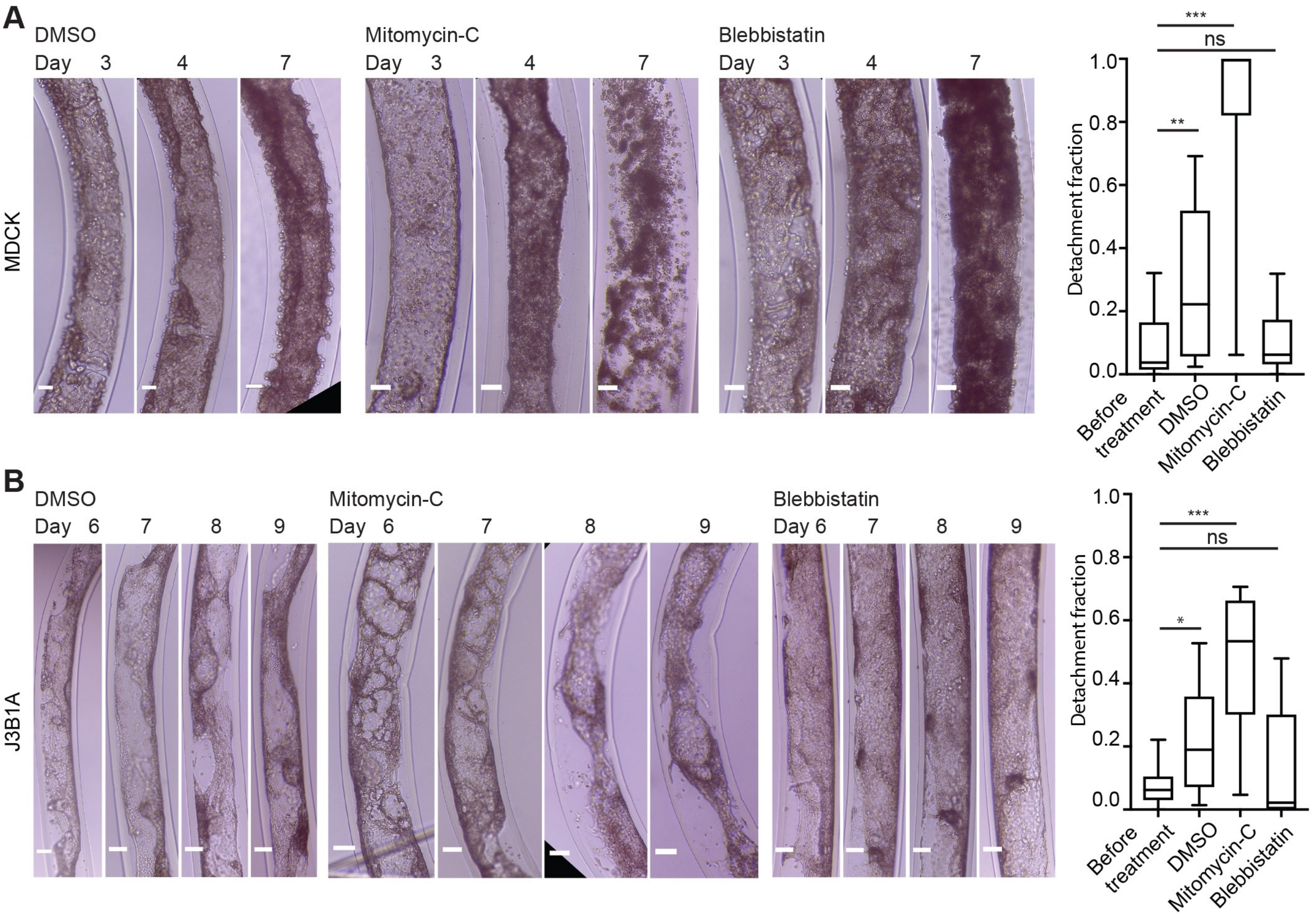

Figure S2. (A-B) Brightfield images of the MDCK (A) and J3B1A (B) tissues growth in alginate tubes under drug treatment. In (A), image of MDCK cells at day 3 and, in (B), image of J3B1A cells at day 6 are obtained just before drug incubation. 30% Matrigel, small tubes. Scale bars: 100 µm. Detachment fraction of MDCK and J3B1A tissues before and from the day after drug tretments (DMSO, Mitomycin-C and Blebbistatin). Comparisons of all conditions before and after treatment (MDCK: DMSO, **, p=0.0066; MitomycinC, ***, p=0.0007; Blebbistatin, ns, p=0.6505; J3B1A: DMSO, *, p=0.0179; Mitomycin-C, ***, p=0.0003; Blebbistatin, ns, p=0.4641, Mann Whitney test, n=7-18 MDCK tubes/condition, n=9-14 J3B1A tubes/condition). Top and bottom of a box indicate 75th and 25th quartiles, respectively; whiskers indicate the min and the max values; the middle line is the median.

Journal of Cell Science • Supplementary information

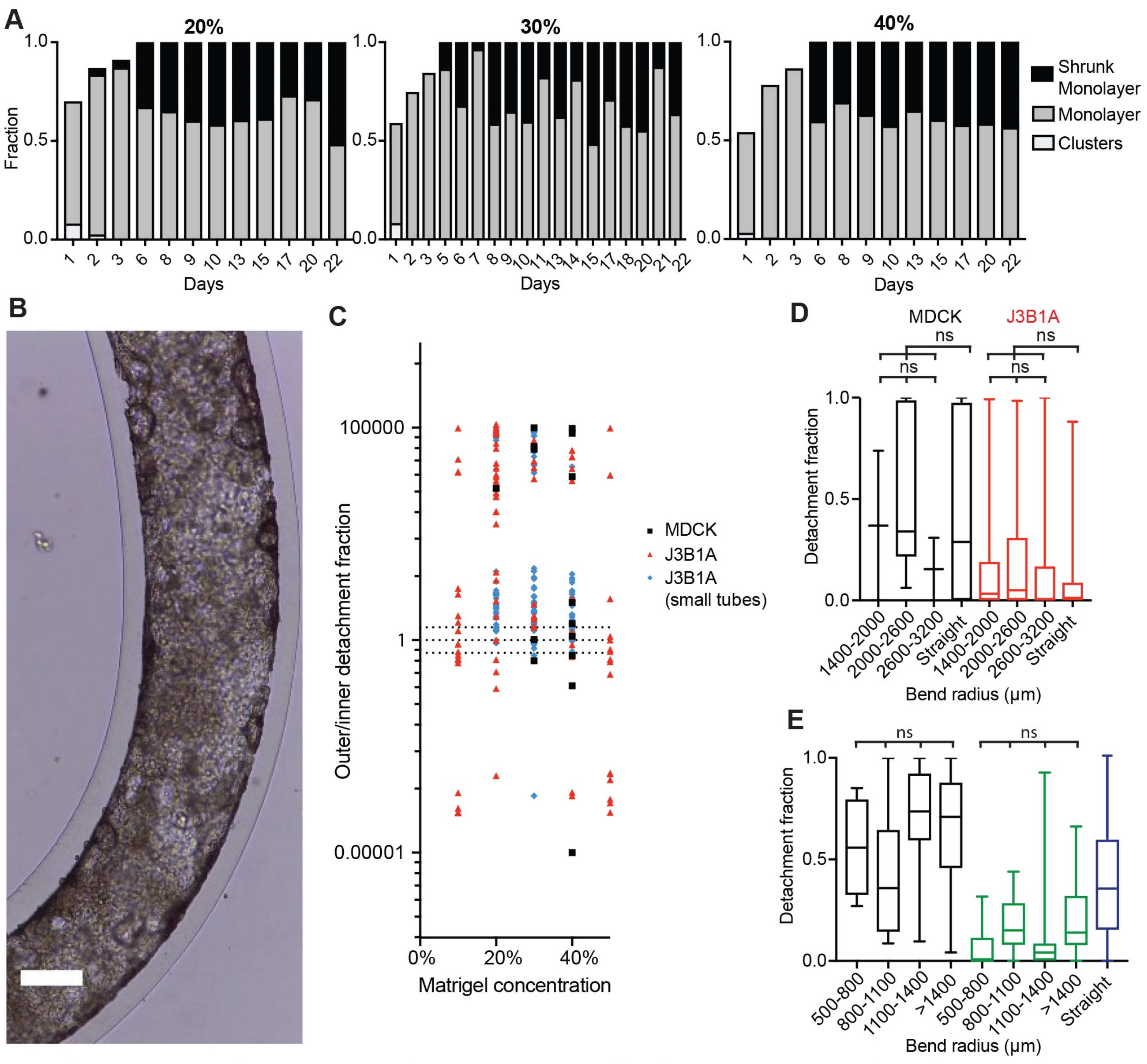

Figure S3. (A) Histograms of the three stages of J3B1A cells in small tubes (20-40% Matrigel). (B) Example of bright field image of a J3B1A tissue with a detachment fration lower than 5% and an extrusion density lower than 0.002 cells/μm (day 7, 50% Matrigel, scale bar: 100μm). (C) Distributions in semi-log scale of the values of the ratio between the detachment fraction of the monolayer on the outer and the inner part of curved tubes, with different Matrigel concentrations (10-50%), for both cell lines (MDCK, black squares; J3B1A, red triangles; J3B1A in small tubes, blue hexagons). Dashed lines are at Y = 0.5; 1; 2. (D) Outer detachment fraction of both cell lines (MDCK, black; J3B1A red) in curved tubes of different ranges of curvature radii of the turn (MDCK: ns, p=0.4476; J3B1A: ns, p=0.6268, Kruskal-Wallis test) compared to straight tubes (MDCK: ns, p>0.9999; J3B1A: ns, p=0.3752, Mann-Whithney test). (E) Detachment fraction at the outer (black, ns, p=0.9328, Kruskal-Wallis test) and the inner (green, ns, p=0.1469) side of curved tubes of J3B1A monolayers in small tubes at different curvature radii of the tube bend. (C-E) n=15 MDCK tubes, n=87 J3B1A tubes, n=127 J3B1A small tubes in C; n=2-45 MDCK tubes/condition, n=14-25 J3B1A tubes/condition in D; n=4-53 J3B1A tubes/condition in E. Data obtained from tubes after day 4 (20-40% Matrigel in D and E). The fraction of detachment is calculated as the ratio between the length of detachment and the overall tissue length. (D, E) Top and bottom of a box indicate 75th and 25th quartiles, respectively; whiskers indicate the min and the max values; the middle line is the median.

Journal of Cell Science • Supplementary information

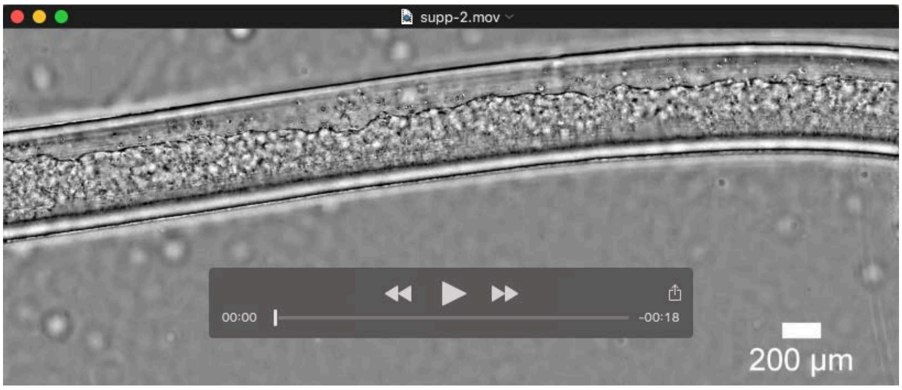

Movie 1. MDCK cell monolayer growth in regular alginate tubes. Phase images acquired with a lens-free microscope from 1 to 6 days after tube formation. Time frame: 10 min. Scale bar: 200 µm.

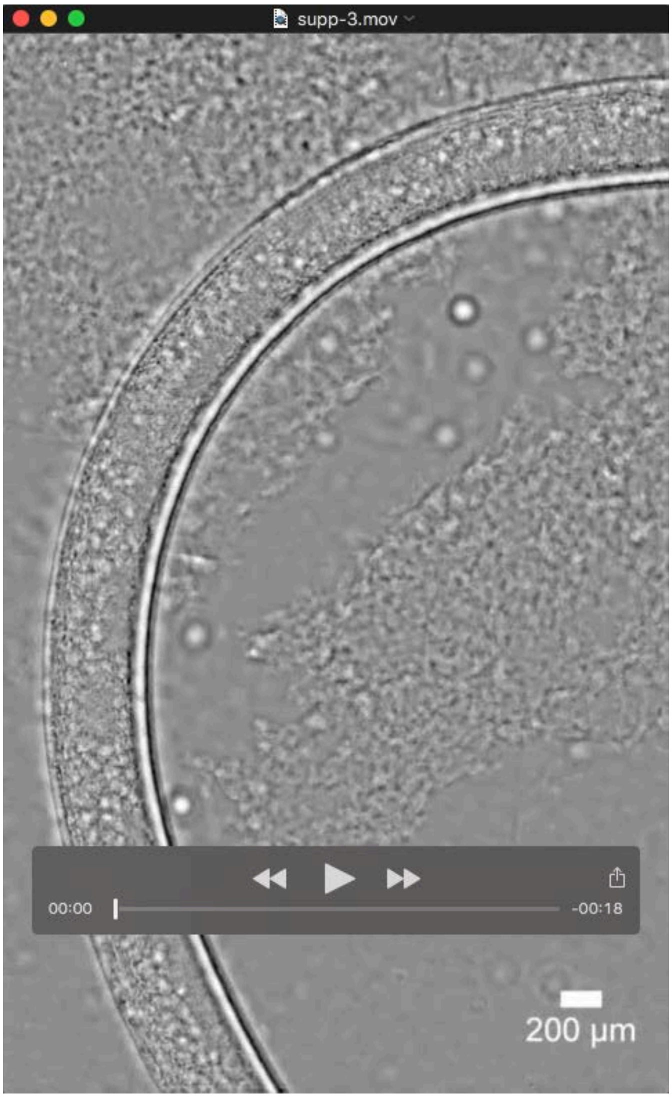

Movie 2. J3B1A cell monolayer growth in regular alginate tubes. Phase images acquired with a lens-free microscope from 1 to 6 days after tube formation. Time frame: 10 min. Scale bar: 200 µm

Journal of Cell Science • Supplementary information
